# Possible Dangers from Salicylic Acid

**Published:** 1877-05

**Authors:** 


### ARTICLE X.

### *Possible Dangers from Salicylic Acid.*

It behooves the admirers of new remedies to be cautious in the use of this acid. M. Blandeau, of Paris, states that, according to dentists, this agent has injurious effects on the teeth. English observers have noticed its effect on the bones, and necrosis of the tibia has been assigned to its use. It evidently possesses considerable affinity for the calcareous salts of bone, as we see the urine loaded with lime salts in an ultra-physiological proportion, from the internal use of the acid. The salicylate of soda presents the same dangers.

If these facts are confirmed, and their verification should be easy, the therapeutical employment of salicylic preparations should be condemned.—*Med. and Surg. Reporter.*

